# Smart Cup for In-Situ 3D Measurement of Wall-Mounted Debris via 2D Sensing Grid in Production Pipelines

**DOI:** 10.3390/mi14020489

**Published:** 2023-02-19

**Authors:** Hao Tian, Sunyi Wang, Minglei Fu, Dayong Ning, Yongjun Gong

**Affiliations:** 1 Naval Architecture and Ocean Engineering College, Dalian Maritime University, Dalian 116026, China; 2 Key Laboratory of Rescue and Salvage Engineering of Liaoning Province, Dalian 116026, China

**Keywords:** integrated sensing grid, soft sensing, soft substrate, smart PIG

## Abstract

The accumulation of separated out impurities from pipeline transported medium onto the pipe wall is a major cause of downtime maintenance of oil and gas production systems. To regularly scrub off wall-mounted debris and probe the severity, pipeline inspection gauges (PIG) are the state-of-the-art tools developed for the task, using the pressure differential across the device as the driving force, and tag-along sensing equipment for wall defects measurement. Currently, the PIG propulsion and sensing tasks are realized by separate compartments, limited to large diameter operations. In this work, a soft solution for medium to small diameter pipelines has been demonstrated. The smart cup with integrated sensing grid is proposed to achieve integrated wall-mounted debris dimensional measurement, without the need of additional sensors. To achieve the goal, this work starts from the mathematical modelling of the geometric problem, to new fabrication procedures, experimental setup, and finally finishes with validation results. Initial results have shown that using the proposed smart cup, the wall-mounted debris can be detected, with modelling error maxed at 5.1%, and deformation detection accuracy between 1.18% and 1.92% with respect to the outer diameter.

## 1. Introduction

### 1.1. Background

**Figure 1:**
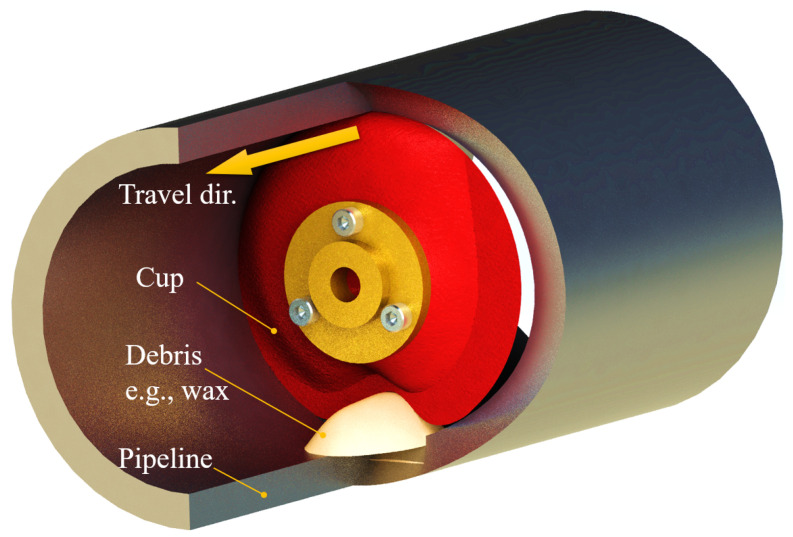
A typical scenario of Φ100 mm PIG travelling in pipeline encountering wall-mounted debris.

Pipeline inspection gauges (PIG) are a critical tool to ensure the operation continuity of oil and gas transportation. The primary function of a PIG is to clear debris and flow obstructing matter, and often it is equipped with sensors designed to inspect structural anomalies or early degradations without halting the entire pipeline transport [1]. The necessity for regular pipeline inspection is due to the suspending fluid state of the transported good inside the pipeline, where separated water, sand, or wax from the transported fossil fuels would condense, and form flow-blocking obstacles, neglecting which would be costly in terms of reduced transportation efficiency or even system failure [2]. In Figure 1, the cross-sectional view of a typical PIG working inside a pipeline is demonstrated. As the key functional component, the rubber cup assumes two main functions during operation: isolating the fluid before and after the PIG to form a contamination control barrier, and creating a pressure differential, which would then become the driving force for the PIG to traverse inside the pipeline, and the force to clean and scrub the debris off the inner wall [3]. So far, most state-of-the-art PIGs with pipeline inspection functions have diameters greater than 300 mm, and the main limiting factor is the bulky size of the inline inspection (ILI) devices. For production pipelines with medium to small inner diameters (ID), there is no practical way of in-situ wall inspection.

The physically isolated working condition of a PIG poses a unique challenge apart from other measurement tasks in more accessible environment. Since most pipelines are made of ferrous material, the enclosed space inside is dead shielded from optical measurement methods, and the high viscosity content such as crude oil would block most laser-based topology mapping methods. Thus, the available sensing technologies left are composed of magnetic flux, ultrasonic, pulsed eddy current, mechanical contact, or acoustic detection [4,5,6]. Tehranchi et al. utilized magnetic flux leakage detection technique to observe and assess the severity of corrosion in pipelines without affecting their operation [7]. Xie et al. found that the magnetic flux leakage detection accuracy is dependent upon the targe material’s magnetic properties [4]. Caleyo et al. achieved high inspection accuracy in controlled environment using acoustic inspection, the post-processing of data requires complex coupled liquid-media models [8]. Through the detection of the coil voltage effect or impedance changes, eddy current and pulse eddy current methods are effective in sensing internal diameter defects and cracks, commonly used for the identification and detection of internal and external diameter of the pipe [9,10], but the applications are limited to conductive materials. By using high-resolution electromagnetic acoustic sensors for internal inspection of pipes, Klann et al. found that electromagnetic acoustic sensing inspection has a unique advantage over other methods for detecting radial and axial cracks in the outer diameter of pipes [11]. However, these sensing methods still face various problems in online pipeline inspection, where the electromagnetic inspection results would be disturbed by the outer defects of pipe as well rather than the targeted inner ones alone. Moreover, the motion-induced eddy currents inside the pipe during high-speed motion prohibit the carrier traversing speed to be limited under 5 m/s [12,13]. Similarly, the carrier speed for the ultrasonic inspection tools is also limited, due to the interfacial wave diffraction and the Doppler Effect, which limit the high speed application. Nonetheless, the commonalities of nearly all the aforementioned sensing technologies require an excitation source and a receiver in pair. To encase all the components, in most commercial PIG’s with inspection functions, they usually take the form of a train with multiple connected cars, where each functional section is contained within one car. Unfortunately, such dimension requirement makes the said sensing technologies infeasible to cases with constrained pipe diameters.

To design more compact PIGs, preferably with integrated inspection functions, one inspiration may be from the soft side. In the past 10 years, as the study of micro electromechanical systems (MEMS) continue to evolve, research on flexible sensors, such as flexible capacitive or resistive displacement, strain, temperature, gas, or optical sensing, have been widely reported. The soft sensors are compact and adaptable to delicate tasks such as health monitoring of vital signs, human–computer interaction [14,15,16]. For example, Lee et al. developed a low-cost flexible pressure sensor based on elastomeric film, which has a fast response time and high sensitivity [15]. Yang et al. studied a flexible capacitive pressure sensor based on ionic liquid, which has a good dynamic response for pressure measurement of surface pressure of complex structures [16]. However, most reported work aimed at human–environment interactions [17]. To allow the flexible sensors to be compatible and applicable with the industrial conditions, environmental compatibility and sensor integration are priorities [18,19].

### 1.2. Proposed Smart Sensing Cup

**Figure 2:**
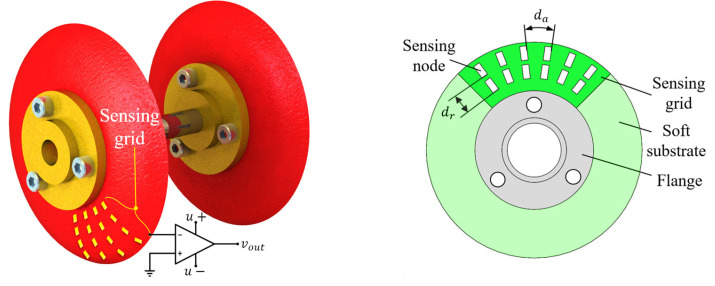
Schematic diagram of the structure of the sensing cup (**left**) and a prototype illustration (**right**).

Facing the aforementioned challenges, we aim at reducing the axial length of the traditional smart PIG devices, and proposed the smart cup solution. The design is shown in Figure 2. Akin to traditional cleaning cups, the proposed device also features a flexible cup, but with an additional sensing grid composed of sensing elements fabricated in 2D patterning method and bounding processes, contrast to a simple molding process traditionally. With the axially driven under pressure differential, the smart cup realizes the common driving function of a traditional cup. However, as the cup substrate makes contact with the debris lodged on the pipe wall, the sensing grid is excited and the dimensional information of the obstacle can be obtained and evaluated. The objective of the research is to validate the functionality of the proposed device, fabricate a prototype, and test out the obstacle measurement accuracy through experiments, in the hope of seeking a new way of a non-invasive pipeline debris sensing method for moderate to small sized pipeline systems.

To achieve its goals, this paper is organized as follows. In Section 2, the mathematical models to evaluate debris size based on the features obtained from the sensing grid is derived, together with the geometric proof for polynomial regression, and the necessary planar interpolation methods. In Section 3, a prototype smart cup is fabricated in-house, with the fabrication process described, followed by the introduction of a PIG testbed with debris emulation capabilities. The initial test results are shown in Section 4, with a few concluding remarks in Section 5.

## 2. Mathematical Models

### 2.1. Debris Crossing Scenario

**Figure 3:**
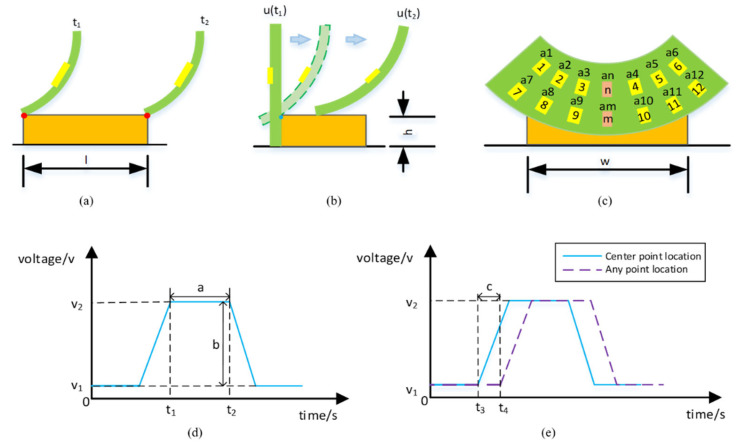
Anticipated features during debris encounter. Note: (**a**,**b**) side views as the cup travels across the orange rectangle debris, (**c**) front view of the cup’s sensing grid w.r.t. the debris, (**d**,**e**) definitions of the anticipated features.

As the cup travels inside a smooth pipeline, the deformation of the cup is expected to be uniform, due to the constant pressure differential across the cup in the axial direction. However, as the circumference of the cup starts to make contact with the debris lodged on the wall, unbalanced deformation will occur. Since the pipe often encloses high pressure fluid, the area of the irregular blockage and deformation of the pipe wall can be a huge threat, which might cause blockage or even rupture. Therefore, understanding the upper and lower boundaries of the debris’ shape can be key to proactive pipeline maintenance. To define the envelope of an object’s three-dimensional space, dimensions along the three Cartesian coordinates are required. If one applies a type of curvature-sensing grid onto the cup surface, to record the localized differences in signal strength, then, the dimensional information of the target debris may be obtained. To do so, one needs sensor feature descriptors to define the correlations with the three orthogonal axes. Assuming the curvature signal from one point of the sensing grid is continuous and differentiable in time, a sudden contact with the debris will be reflected similarly as a hump in the undisturbed signal, where the disturbed duration may be correlated to the length of the debris, while the strength of the signal reflects the debris height, and the time difference among different sensing units in the grid can be used to determine the width of the debris, as illustrated in Figure 3. In this way, the magnitudes of length (*L*), width (*W*) and height (*H*) of the foreign object can be obtained, through evaluating the temporal features from the expected outputs of the sensing grid. The features are defined in Table 1.

### 2.2. Disturbance Vector and Debris Sizing

**Figure 4:**
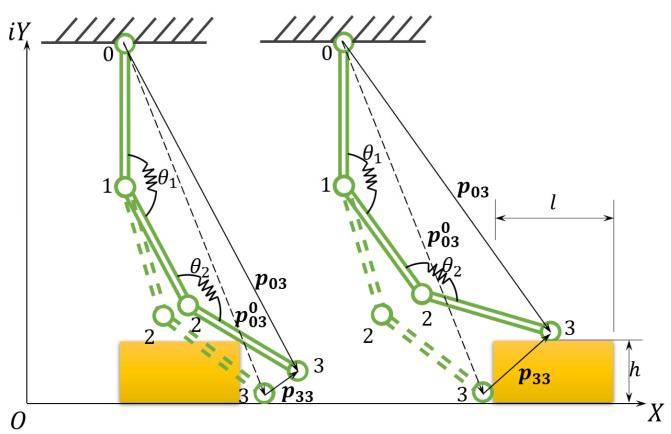
Schematic diagram of debris block traversing. Note: (**Left**) cup starts to make contact, (**Right**) cup makes full contact with the block. The cup is travelling from right to left, w.r.t. the set block.

If no debris exists in the traversal direction of the cup, the cup should experience constant deformation, if the surface roughness, travel speed or flow pattern do not affect the cup dynamic equilibrium. In this work, the cross section of the cup is assumed to be 1D, and the cup is assumed to be sectionally rigid, where the rigid sections are connected by flexible joints. A disturbance vector, $p_{jj}$, is defined, to capture the deviation of the outer rim (as the tip 3 in Figure 4) from the non-disturbed case. A special case of three sections with two joints is shown in Figure 4.

In the vector loop shown in Figure 4, the disturbance vector $p_{33}$ can be obtained from the vector loop relationship.

(1) $$p_{33}=p_{03}-p_{03}^{0}$$

where the super script 0 denotes the undisturbed positional vector (without debris), the subscripts *ij* denotes the vector originates from *i*, and points to *j*, and the bold format denotes a vector. To reconstruct the debris in 3D space, the three key dimensional values (*xyz*) are the required minimums. According to the illustration in Figure 3, the length and height data can be obtained by $p_{33}$ alone, as according to Euler’s Formula, the planar vector contains both the *x* and *y* information, if $p_{33}$ were to be solved.

(2) $$p_{33}=p_{33}e^{i\theta_{33}}=p_{33}\cos\theta_{33}+i\sin\theta_{33}$$

To solve, we first expand the vector loop down to a summation of the constitutive single vectors.

(3) $$p_{33}=p_{01}+p_{12}+p_{23}+p_{01}^{0}-p_{12}^{0}-p_{23}^{0}$$

Since the cup is mechanically clamped to the central flange, it is therefore safe to assume the first vector, $p_{01}$, is constant. Then, the subsequent the sections/links can be viewed as a displacement and rotation from the original.

(4) $$p_{12}=p_{01}+p_{01}R_{01}=p_{01}\left(1+e^{i\theta_{01}}\right)$$

where $R_{01}$ = $e^{i\theta_{01}}$ is the rotational operator.

If we assume the constant radius of curvature along the radial direction of the cup, and plug Equation (4) into Equation (3), we have

(5) $$p_{33}=p_{01}R_{01}^{2}+3p_{01}R_{01}+2p_{01}-p_{12}^{0}-p_{23}^{0}$$

Observe Equation (5), since $p_{01}$, $p_{12}^{0}$, $p_{23}^{0}$ are constants by definition, it is clear that $p_{33}$ takes the form of a second order polynomial of $R_{01}$. In a more general form, and if the power of the rotational operator were to be higher than 2 (i.e., more sections present other than only 3 as in Figure 4), the disturbance vector can be expressed as a function of excitation $Z_{i}$.

(6) $$p_{jj}=\left\{\beta_{1}Z_{i}^{j}+\beta_{2}Z_{i}^{j-1}+\cdots +\beta_{j+1}\right\}$$

### 2.3. Sensing Grid Data Interpolation

**Figure 5:**
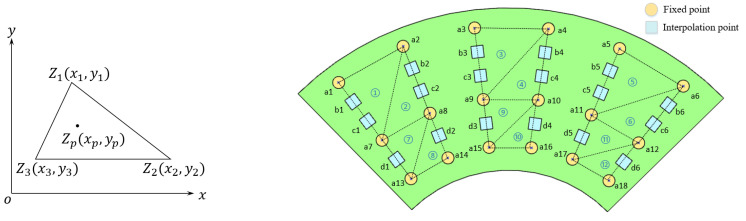
Schematic diagram of planar data interpolation. Note the numbers in circles mark the triangles for interpolation operations, treating the fixed points as known.

The proposed smart sensing cup employs a sensing grid design where each sensing node can evaluate the deformation curvature in-situ, if evaluates all the nodes with the feature models, the overall curvature information as well as the displacement can be found. Since only finite nodes can be deployed on the cup, the sensing values for the vacant space in between can only be obtained via interpolation. The particular interpolation problem on the sensing cup is shown in Figure 5.

For a triangle in a 2D Cartesian coordinate system, if $Z(x_{p},y_{p})$ corresponds to the strength of a smooth and differentiable signal at point on a plane, it naturally satisfies the following polynomial:

(7) $$Z(x_{p},y_{p})=\alpha_{1}+\alpha_{2}x+\alpha_{3}y$$

To obtain the coefficients, a simultaneous equation taking the signal strength at the apexes of the triangle can be used.

(8) $$\left\{\begin{array}{c} Z_{1}\\ Z_{2}\\ Z_{3} \end{array}\right\}=\left[\begin{array}{ccc} 1 & x_{1} & y_{1}\\ 1 & x_{2} & y_{2}\\ 1 & x_{3} & y_{3} \end{array}\right]\cdot \left\{\begin{array}{c} \propto_{1}\\ \propto_{2}\\ \propto_{3} \end{array}\right\}$$

Through matrix inversion, the coefficient vector ⌈∝⌉ is found.

(9) $$\lceil \propto \rceil =G^{-1}Z$$

and,

(10) $$G=\left[\begin{array}{ccc} 1 & x_{1} & y_{1}\\ 1 & x_{2} & y_{2}\\ 1 & x_{3} & y_{3} \end{array}\right]$$

From the above equations, any signal value within the defined triangle can be evaluated.

## 3. Experimental Design

### 3.1. Smart Cup Prototype Preparation

**Figure 6:**
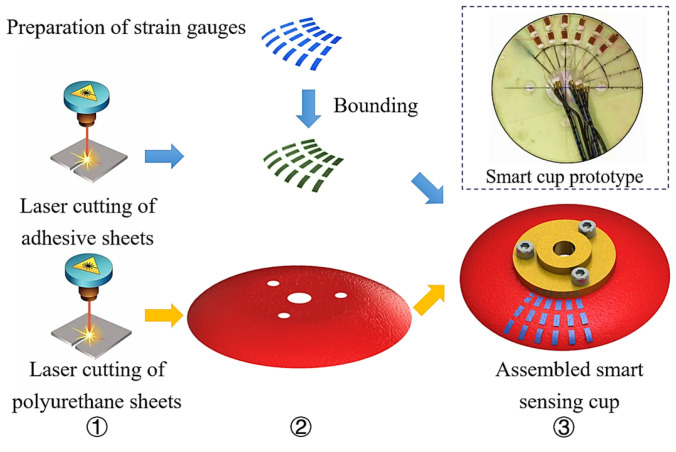
Fabrication procedures for the smart cup.

**Figure 7:**
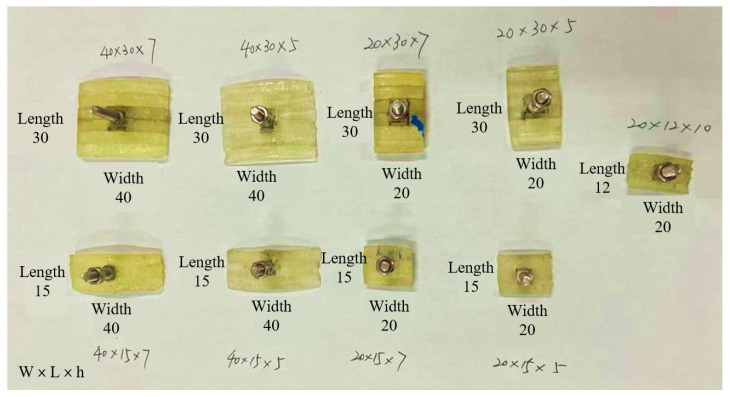
Assembled mock debris blocks for the smart cup test (unit in mm).

Traditional cups are made of rubber materials, which are not compatible with the proposed soft sensing grid design. To achieve the smart cup functionality, a new 2D patterning fabrication method is developed. To validate the function and performance of the proposed smart cup, a prototype with 12 sensing nodes using micro strain gauges was fabricated, with an effective resolution of a 72-node grid if a full sensitive cup were to be implemented. The preparation of the prototype smart cup with sensing grid was done in lab. An illustration of the sensing cup preparation steps is shown in Figure 6. An economical table-top lase cutter model was employed, the fabrication was carried out at 80 W and the feed speed was set at 1.4 mm/s. The fabrication process starts from the laser lithography of polyurethane sheet, and adhesive sheets, followed by sensing elements lamination with the soft substrate, and finishes at the electrical sensing wire bounding. It is worth mentioning that, at current stage, the sensing nodes (micro strain gauges) were bounded to the polyurethane substrate manually. In the future, these steps can be further automated if new sensing elements were used (e.g., polyvinylidene fluoride sheets with physical vapor deposited piezoelectric crystals). Additionally, a set of mock debris were also fabricated in lab with similar procedures to the cup, as demonstrated in Figure 7, and the dimension details can be found in Table 2.

### 3.2. Testbed Design

**Figure 8:**
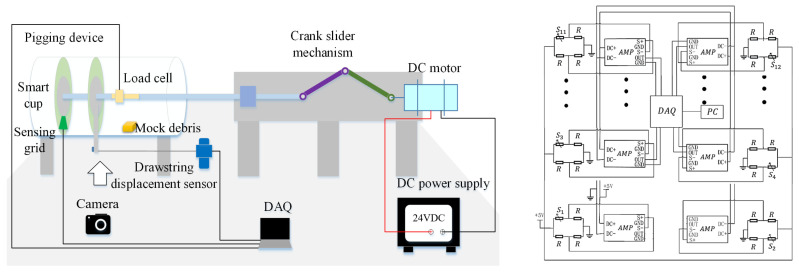
Schematic diagram of the test platform (**left**) and the parallel signal processing circuit design (**right**).

**Figure 9:**
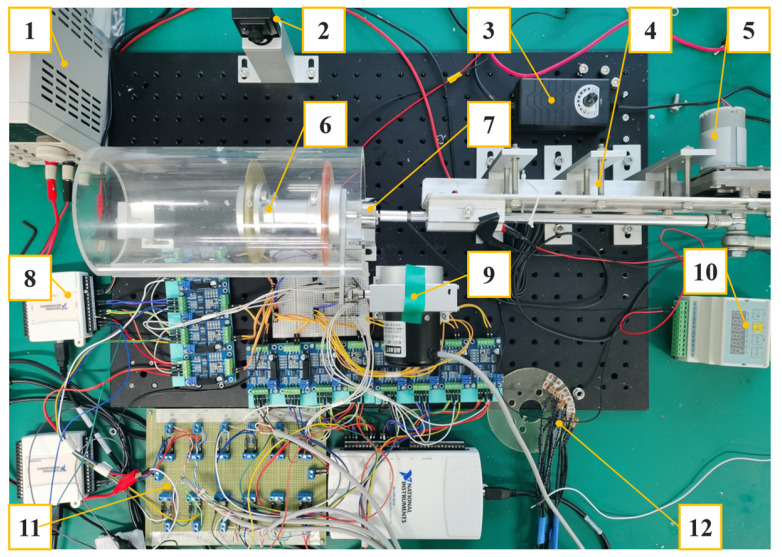
A photo of the smart cup test bed. Note: 1. DC power supply, 2. Digital high shutter speed camera, 3. Motor regulator, 4. Slider-crank mechanism, 5. DC motor, 6. Φ100 PIG with smart cup, 7. Load cell, 8. DAQ (Data acquisition unit), 9. Drawstring displacement sensor, 10. Load cell transducer, 11. Bridge and amplifier circuit, 12. Spare smart cup.

To emulate the working environment of the PIG, a Φ100 mm test bed was designed and assembled in the lab. The experiment bench device is composed of a crank slider driving mechanism, a smart cup prototype, a transparent pipeline, and a mount for installing different sized debris for testing. The operation principle of the testbed is shown in Figure 8. A crank-slider mechanism, driven by a DC motor was used to emulate the pressure differential driving force to realize the translational motion of the prototype. A load cell was mounted on the axial with a range of 0–500 N and an accuracy of 0.2%. A drawstring displacement sensor with range 0–350 mm, accuracy 0.15% was used to determine the motion and displacement of the smart cup. The data signals were collected via a data acquisition unit (DAQ). As for the prototype smart cup, a strain gauge grid of 12 nodes was laminated on prototype cup substrate, where each node was equipped with a bridge and an amplifier circuit for signal differentiation and amplification. Each bridge circuit was composed of 3 120 Ω fixed-value resistors and 1 120 Ω strain gauge. The bridge and the amplifier circuits were powered by two 5 V DC power supplies to avoid common mode interference. Before the general experiment, each bridge and the corresponding amplifier was calibrated to a common zero with same amplification factors. In addition, the relationship between the primary (x-axis) displacements of the strain gauges versus output voltages were also obtained using camera data. The sampling frequency of the acquisition device was 2 kHz, and the duration of each acquisition was 5 s. A photo of the testbed is shown in Figure 9, and the rest of the specifications can be found in Table 3.

## 4. Results and Discussions

### 4.1. Prototype Function Validation

**Figure 10:**
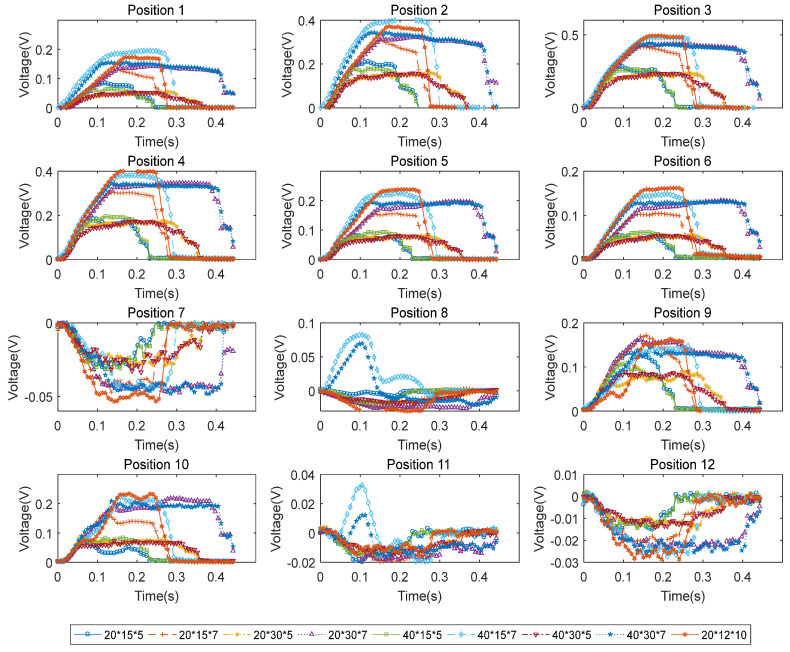
Output voltage signals of No. 1–12 strain gauges when crossing over different mock debris blocks. Note: the bottom legends denote the size of the block, in length by width by height in mm.

A set of experiments with different mock debris blocks were tested to demonstrate performance of the prototype smart cup. For specific mock debris block, five groups of tests were carried out to reduce random error. During each experiment, the test bed was controlled to run only one motion cycle (a clockwise 2π rad rotation of the crankshaft from its set zero location). The driving starting position and driving power of the crank-link mechanism driving device were consistent among the tests, so as to ensure that the sensing grid was subjected to the same force at the moment of contacting the obstacle block. The output voltage signals of No. 1–12 strain gauges passing through different obstacles are shown in Figure 10. Obviously, as the smart cup crosses over different sized obstacles, the corresponding voltage signals from the sensing grid vary as well; also, the signal strength values are distinctive enough from case-to-case to allow for further dimensional determination tasks, which all validate the basic functions of the proposed device.

### 4.2. Features Analysis

**Figure 11:**
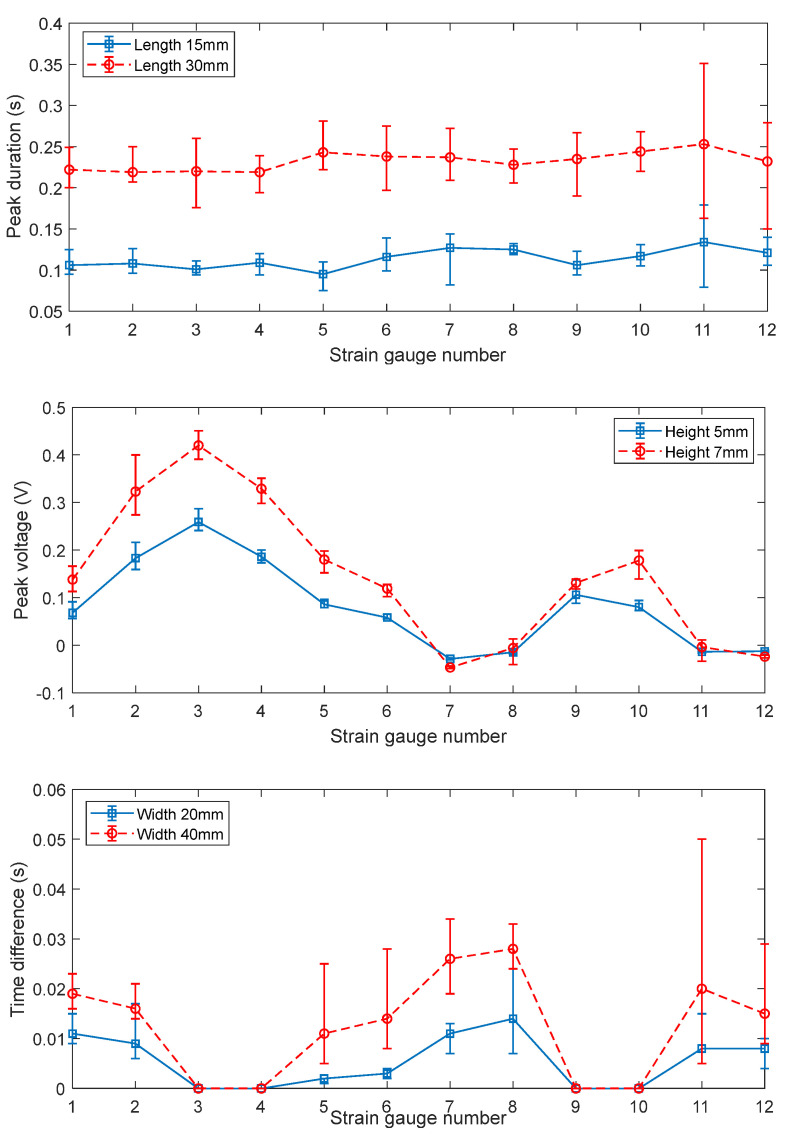
Features analysis. Note: (**top**) signal peak duration, (**mid**) peak voltage, and (**bottom**) signal initiation time difference referenced to the central positions (m, n in Figure 3). The error bar denotes the bounds that contain 5 separated tests under the same condition.

The preliminary analysis of the sensing grid’s signal output only shows simple trends. To evaluate the signals obtained from the experiments, the features of the signals were computed, which were the signal peak duration, the peak magnitude, and time difference of the initial voltage changing point of the strain gauge at different positions to that at the central positions (m, n in Figure 3). The signal peak duration at each sensing node is plotted in Figure 11 (top). From the figure, it can be found that the signal peak duration is sensitive to the length of the mock debris block. It is as expected since according to Equation (6), if no excitation (deformation) presents, the disturbance vector reduces to a constant. So, the signal peak duration, or the energized duration can be viewed as a key feature to be evaluated to obtain the length information of the block. The middle plot in Figure 11 shows the peak magnitudes of the signals as the smart cup traverses different debris blocks. It is found that the signals can be differentiated as the heights of the blocks vary. Interestingly, the results show clearly repetitive patterns if comparing the strain gauges of 1–6 to 7–12, where the No. 1–6 corresponds to the inner while the 7–12 to the outer rows. This is expected since the strain gauges of 1–6 and 7–12 are located concentrically. If looking closely, the signal strength at the outer circle is lower than that at the inner, the attenuation of the signal strength at the outer rim can be attributed to the localized bending of the cup. From the observation of feature 3 in Figure 11 (bottom), the signal initiation time differences of the front row (No. 1–6) compared to No. 3 or 4 (depending on which is the nearest), and of the back row (No. 7–12) compared to No. 9 or 10, are found to be increasing with the distance from the reference point. This trend is in line with the layout of the sensing grid, since the part of the cup that comes in contact with the debris block first would always be the center, as the cup travel further, then, the materials away from the center start to experience deformation.

**Figure 12:**
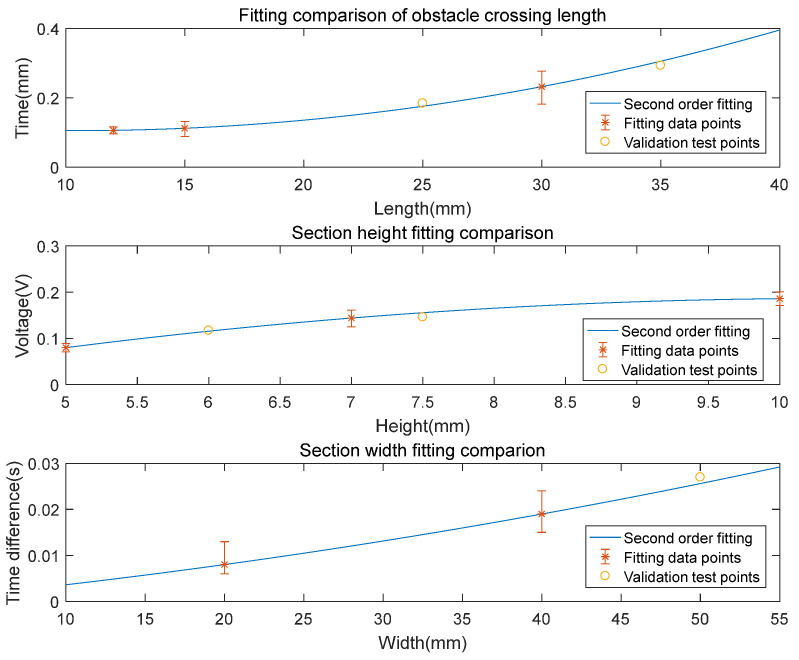
Debris dimension regression results and validation.

The results in Figure 11 have demonstrate the selected features can be used as the excitations for the disturbance vector calculation. Since in the current experiment, only two rows of sensors were implemented, the order for the Equation (6) is set to 2. Based on the features definitions in Table 1, quadratic regression operations of the three key dimensional factors were conducted, the resulted coefficients can be found in Table 4. Based on the regression coefficients, the debris block’s dimension regression results are plotted and validated in Figure 12. The results have demonstrated good second order fitting of the experimental data points. By testing out the three regression functions against a new set of experiments, the maximum error between the model and experiment is less than 5.1%, and the regression results are believed to be reasonable.

### 4.3. Debris Shape Visualization

**Figure 13:**
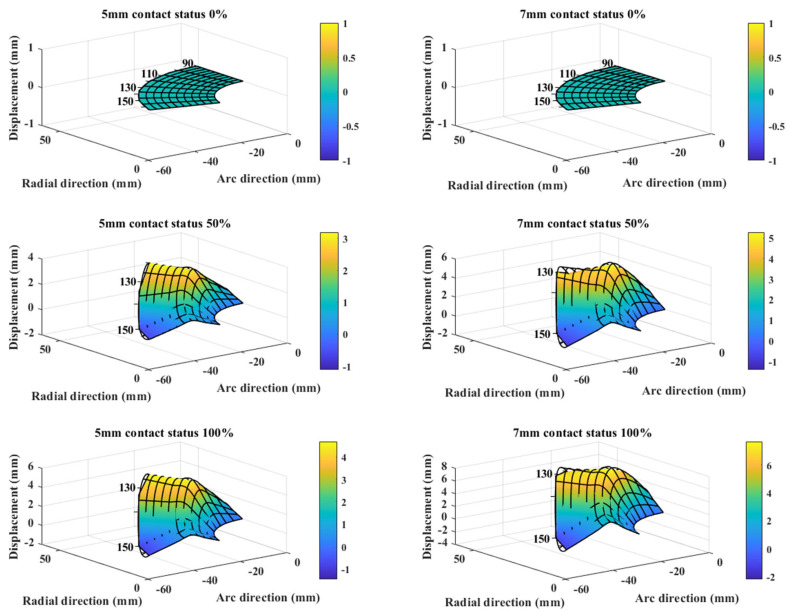
Displacement map of the smart cup at each sensing node during different stages of debris block crossing.

With the dimension parameters of the debris block obtainable with the proposed smart cup, efforts were made to visualized the measured debris. Figure 13 illustrates the sensing grid displacement map as the prototype crosses over the debris blocks of 5 mm or 7 mm height. Since the prototype sensing grid has only 12 measurement points, the signal magnitudes at the blank space were interpolated using Equation (7). Obviously, when the cup is not in contact with the debris block, no deformation is found, as the cup makes further contact with the block, the displacement continues to increase. Same trends were observed for both the 5 mm and 7 mm cases. Specifically, as the height of the debris block increases from 5 mm to 7 mm, the maximum displacement value in the 50% contact case increases from 3.1 mm to 5.3 mm, and for the 100% case, from 4.5 mm to 7.7 mm.

**Figure 14:**
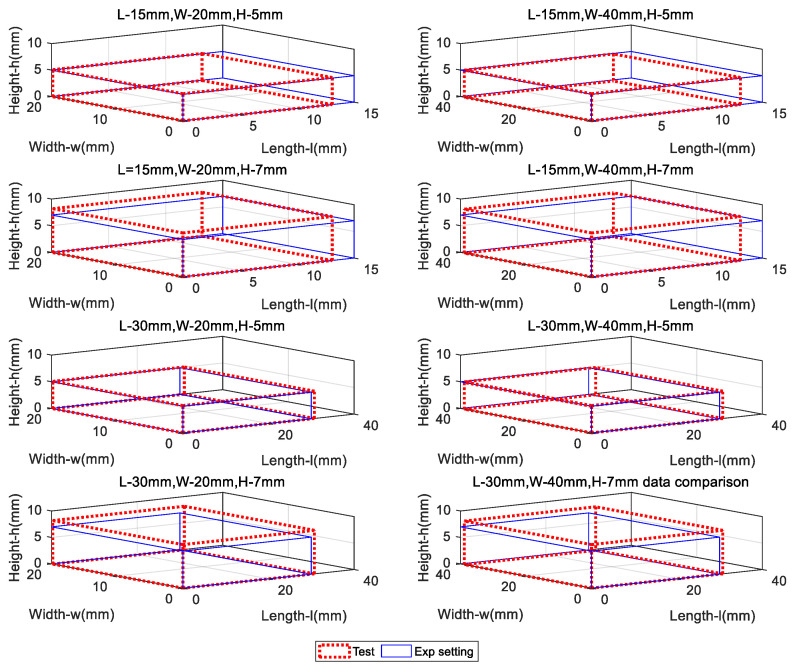
Comparison of visualized mock debris block shapes between the test groups and experimental settings.

Finally, Figure 14 visualizes the measured debris dimensions as the prototype cup crosses over a selection of mock debris blocks. The proposed device and measurement method has demonstrated good consistency between the measurement results and the experimental settings. The maximum deviation of the tested model from the experiment or the absolute measurement errors of the height, length, and width are found to be 1.23 mm, 1.92 mm and 1.18 mm, respectively. If compared with the outer diameter (OD), the detection accuracy can be found to be between 1.18% (OD) and 1.92% (OD), which can be comparable in terms of performance with the state-of-the-art market products such as the TDW’s high resolution deformation measurement tool for smart PIG’s [20]. Moreover, it was found the accuracies in the three orthogonal directions are around the same magnitude, suggesting the features designed in Table 1 allow for accurate and balanced detection of pipe debris’ boundaries. However, it is worth mentioning that this result was conducted under laboratory condition using squared surfaced mock debris blocks, and without actual interferences such as the pressurized environment and transient flow fields. Nonetheless, the initial performance of the proposed device has been demonstrated and potential application can be promising. In the future, more detailed testing and under near-production-line conditions experiments will be conducted to further study the characteristics of the smart cup with 2D sensing grid.

## 5. Conclusions

In this work, a soft solution for medium to small diameter pipeline debris measurement was demonstrated. A smart cup with integrated sensing grid was proposed to achieve wall-mounted debris block dimensional measurement with the prototype alone, without the need of additional tag-alone devices. To achieve the goal, this work starts from the mathematical modelling to experimental setup, and finally finishes with preliminary validation results. The key finding of the paper are:

1. The function of inline inspection of pipeline defects can be integrated into the proposed smart cup, to achieve driving and inspection at the same time, allowing for the extremely compact design of next generation smart PIGs.

2. New fabrication procedures specially designed for the smart cup are presented, where 2D patterning technologies are applied to the soft sensor assembly.

3. Using the proposed smart cup, the wall-mounted debris can be detected, with modelling error maxed at 5.1%, and deformation detection accuracy between 1.18% and 1.92%, with respect to the outer diameter.

So far, the proposed functions have been validated. In future work, a prototype with full sensing array may be constructed, and the debris measurement algorithm can be further improved to allow for finer details reconstruction.

## Figures and Tables

**Table 1:** Debris feature definitions (for *j*th sensing element, with total grid size of N).

Features Definitions	Formula
Debris cross section length -signal peak duration	$Z_{1}=\frac{1}{N}\overset{N}{\underset{j=1}{\sum }}\left(t_{2}^{j}-t_{1}^{j}\right)$
Debris cross section height -signal maximum magnitude	$Z_{2}=\frac{1}{N}\overset{N}{\underset{j=1}{\sum }}u_{max}\left(t\right)$
Debris cross section width -differences of the signal initiation time	$Z_{3}=\frac{1}{N}\overset{N}{\underset{j=1}{\sum }}\left(t_{4}^{j}-t_{3}^{m,n}\right)$

**Table 2:** Debris block feature size chart (unit in mm).

Block No.	1	2	3	4	5	6	7	8	9
Width	20	20	20	20	40	40	40	40	20
Length	15	15	30	30	15	15	30	30	12
Height	5	7	5	7	5	7	5	7	10

**Table 3:** Experimental data sheet.

Parameter Name	Value	Unit
Substrate diameter	96	mm
Substrate thickness	2	mm
Substrate material	Polyurethane	-
Displacement range	30–150	mm
Number of amplifiers	12	-
Sensor node count	12	-
Amplification factor	80	-

**Table 4:** Regression coefficients for the disturbance vector (debris dimensions).

Feature Designations	$\mathrm{Coeff}.\;1\;(\beta_{1})$	$\mathrm{Coeff}.\;2\;(\beta_{2})$	$\mathrm{Coeff}.\;3\;(\beta_{3})$
Debris length	$3.33\times 10^{-4}$	$-7.0\times 10^{-3}$	0.142
Debris width	$-3.6\times 10^{-3}$	$7.52\times 10^{-2}$	−0.206
Debris height	$3.75\times 10^{-6}$	$3.25\times 10^{-4}$	0

## Data Availability

The data supporting reported results can be made available via requesting the corresponding author.
